# Recovery of *Mycobacterium tuberculosis* Complex Isolates Including Pre–Extensively Drug-Resistant Strains From Cattle at a Slaughterhouse in Chennai, India

**DOI:** 10.1093/ofid/ofae733

**Published:** 2024-12-19

**Authors:** Harini Ramanujam, Ahmed Kabir Refaya, Kannan Thiruvengadam, Natesan Pazhanivel, Devika Kandasamy, Ashokkumar Shanmugavel, Ammayappan Radhakrishnan, Golla Radhika, Rajkumar Ravi, Neelakandan Ravi, Maheswaran Palanisamy, Sivakumar Shanmugam, Tod P Stuber, Vivek Kapur, Kannan Palaniyandi

**Affiliations:** Department of Immunology, ICMR–National Institute for Research in Tuberculosis, Chennai, India; Department of Immunology, ICMR–National Institute for Research in Tuberculosis, Chennai, India; Department of Statistics, ICMR–National Institute for Research in Tuberculosis, Chennai, India; Department of Pathology, Madras Veterinary College, Chennai, India; Department of Bacteriology, ICMR–National Institute for Research in Tuberculosis, Chennai, India; Department of Bacteriology, ICMR–National Institute for Research in Tuberculosis, Chennai, India; Department of Bacteriology, ICMR–National Institute for Research in Tuberculosis, Chennai, India; Department of Bacteriology, ICMR–National Institute for Research in Tuberculosis, Chennai, India; Department of Bacteriology, ICMR–National Institute for Research in Tuberculosis, Chennai, India; Corporation Slaughterhouse, Greater Chennai Corporation, Chennai, India; Corporation Slaughterhouse, Greater Chennai Corporation, Chennai, India; Department of Bacteriology, ICMR–National Institute for Research in Tuberculosis, Chennai, India; National Veterinary Services Laboratories, US Department of Agriculture, Ames, Iowa, USA; Huck Institutes of the Life Sciences, Pennsylvania State University, University Park, Pennsylvania, USA; Department of Immunology, ICMR–National Institute for Research in Tuberculosis, Chennai, India

**Keywords:** India, *Mycobacterium tuberculosis* complex, One Health, pre-XDR tuberculosis, zoonosis

## Abstract

**Background:**

India has the highest global burden of human tuberculosis (TB) and the largest cattle herd with endemic bovine TB (bTB). However, the extent of cross-species transmission and the zoonotic spillover risk, including drug-resistant *Mycobacterium tuberculosis* complex (MTBC) strains circulating in cattle, remain uncharacterized.

**Methods:**

To address this major knowledge gap, we investigated tissue samples from 500 apparently healthy cattle at a slaughterhouse in Chennai, India. Whole genome sequencing was performed to characterize the isolates.

**Results:**

Sixteen animals (32 per 1000 [95% confidence interval, 16–47]) were MTBC-positive, a rate that is nearly an order of magnitude greater than the reported human TB incidence in the region. Thirteen isolates were identified as *Mycobacterium orygis* and 3 were *M tuberculosis*: 1 was a mixed infection of *M tuberculosis* lineage 1 and *M orygis*, and the other 2 had pure growth of *M tuberculosis* lineage 2, in both cases pre–extensively drug-resistant (pre-XDR) with identical resistance patterns and separated by 7 single-nucleotide polymorphisms. The results confirm that bTB in this region is primarily due to *M orygis* and *M tuberculosis*, and not *Mycobacterium bovis*.

**Conclusions:**

The detection of pre-XDR *M tuberculosis* in cattle highlights a potential public health concern, since controlling human TB alone may be insufficient without addressing bovine TB. Overall, our findings underscore an urgent need for targeted interventions to mitigate zoonotic tuberculosis transmission in regions where bTB is endemic.

## Teaser

In India, zoonotic tuberculosis spillover risks remain poorly understood. This study identified *Mycobacterium orygis* and pre-XDR *Mycobacterium tuberculosis* in Chennai cattle, underscoring the need for targeted interventions to manage cross-species transmission and prevent zoonotic TB spread from cattle to humans.

Bovine tuberculosis (bTB), a chronic infection of bovines and other mammals caused by members of the *Mycobacterium tuberculosis* complex (MTBC), is a significant source of zoonotic tuberculosis (zTB) [1]. Given that specific transmission pathways of MTBC members remain to be fully elucidated, we adapt the pragmatic definition of zTB as proposed by Duffy et al, as tuberculosis (TB) disease in humans, diagnosed based on epidemiological evidence of relevant animal or environmental exposure and/or microbiological confirmation of an MTBC subspecies commonly found in animals [2]. In 2019, an estimated 140 000 zTB cases were reported, underscoring the disease's major zoonotic threat and its considerable public health implications and a risk to the successful implementation of the End TB strategy in regions where TB is endemic in both humans and animals [3]. This is because TB control programs that focus solely on reducing human-to-human transmission will be inadequate if cattle remain a reservoir for spillover risk [4]. Therefore, it is increasingly recognized that addressing zTB requires a comprehensive approach that includes effective monitoring and control of bTB in livestock to mitigate the risk of zoonotic transmission.

India accounts for 27% of the global TB burden, with 2.4 million reported TB cases in 2022 and a prevalence of 312 per 10 0000 population in 2021. This includes 63 801 cases of multidrug-resistant (MDR) and rifampicin-resistant TB and 12 002 cases of pre–extensively drug-resistant (pre-XDR) TB, that is, TB strains that are resistant to not only rifampicin and isoniazid, but also resistant to any fluoroquinolone while still being susceptible to other second-line injectable agents [5–8]. Concurrently, the prevalence of bTB in India is estimated at 7.3%, affecting approximately 21.8 million cattle and buffalo [9]. Zoonotic TB is often associated with extrapulmonary and pediatric infections, which are challenging to diagnose due to diverse clinical presentations, difficulty in obtaining appropriate samples, and the paucibacillary nature of the disease [10]. This complicates accurate assessment of the true burden and risk, hindering targeted interventions and control strategies for India's National TB Elimination Program and the global End TB goals by 2035. For example, *Mycobacterium orygis*, a pathogen within the MTBC belonging to the animal lineage, has gained recognition as a cause of TB in humans, particularly in South Asia. Clinical manifestations of *M orygis* infections are presenting as both pulmonary and extrapulmonary TB. In a study conducted in South India, all 8 confirmed cases of *M orygis* involved extrapulmonary disease, with 2 cases also showing pulmonary involvement [11]. Alternatively, a study in Canada reports that 10 of 21 cases showed pulmonary involvement, whereas 5 extrapulmonary cases were reported, and 6 cases of *M orygis* involving both. The demographic profile of *M orygis*–affected individuals indicates a higher prevalence among older females, particularly those originating from South Asia, with up to 90% of *M orygis* cases seen in women [12]. Additionally, the absence of a coordinated national bTB surveillance and control program in India exacerbates this issue, further highlighting an urgent unmet need for comprehensive surveillance to accurately assess and address the burden and risk of zTB in India.

Abattoir-based slaughter surveillance is a well-recognized passive strategy for assessing the level of bTB in livestock. In regions of the United Kingdom that have endemic bTB, 18%–28% of new bTB herd breakdowns are first detected through slaughterhouse surveillance, underscoring its importance in bTB control, especially in endemic areas [13]. However, even though the global prevalence of visible lesions (VLs) in slaughtered cattle is estimated at 426 per 1000, not all VL-positive animals are infected with MTBC, and not all MTBC infections result in VLs [14]. This highlights the need for unbiased tissue sampling from slaughtered cattle to accurately assess bTB prevalence. In India, 2 recent exploratory studies in the northeastern region of the country reported a notable (∼13%) prevalence based on slaughterhouse surveillance [15, 16]. However, these studies provided no information on the circulating MTBC lineages, and it is unclear if these findings are representative of the broader situation in the country. This is important because recent findings, including our own work, have shown that bTB in India results primarily from *M orygis*, not *Mycobacterium bovis*, prompting a reconsideration of the definition and causes of zTB in this region [17, 18].

Hence, given the paucity of information on passive slaughterhouse surveillance and molecular characterization of MTBC among cattle in India, we sought to assess the prevalence and characterize MTBC isolates recovered through passive slaughter surveillance in Chennai, a large metropolitan area in Tamil Nadu, southern India. By performing bacterial culture, drug susceptibility testing (DST), and whole genome sequencing (WGS), we determined the proportion of infected animals and identified the MTBC lineages circulating among cattle and their drug susceptibility profiles. Our comprehensive approach helped provide key missing insights into bTB burden and highlights the zoonotic risk including from pre-XDR organisms recovered from cattle in India. Together, the findings strongly support an urgent need for the development of effective bTB and zTB control strategies to help realize the global efforts to end TB and safeguard public health.

## MATERIALS AND METHODS

### Study Site and Sample Collection

The samples for this cross-sectional study were collected from the Greater Chennai Corporation slaughterhouse in Perambur, Chennai, India, with requisite permits from the City Health Officer, Public Health Department. In Tamil Nadu, the slaughter of cows is prohibited; therefore, a mixture of native and crossbred male bovines, including bulls, bullocks, and bull calves from Chennai, its outskirts, and the southern parts of Andhra Pradesh are slaughtered at this state-sanctioned facility. This slaughterhouse is a major source of meat for Chennai and surrounding areas. Approximately 50 male bovines are processed each weekday, and between 80 and 100 are processed during weekends.

A total of 567 samples were collected from 500 animals over a period of 3 years. A convenience sampling method was used, with no attempts to select animals. This pragmatic approach, though not optimal for estimating true prevalence, was appropriate for our primary objective of characterizing the molecular epidemiology of mycobacterial strains circulating in cattle in this region. This sampling method was necessitated by logistical and resource limitations. Bronchial and mediastinal lymph node samples, as well as other tissue samples with or without suspected TB lesions, were collected by the same person using sterile scissors and forceps from slaughtered cattle in sterile 50-mL screw-cap containers that were sealed on site and brought to the facilities at the Indian Council of Medical Research–National Institute for Research in Tuberculosis within the hour and stored at −80℃ until further processing.

### Sample Processing and Culture

The samples were retrieved from −80℃ and thawed on ice. To limit surface contamination, a saline dilution method was used [19]. After trimming fat tissues, samples were placed in 1:200 dilution of 8.25% hypochlorite bleach solution for 15–30 minutes, rinsed with 0.9% saline, cut into small pieces, and homogenized using a Fast-Prep 24–5G instrument (MP Biomedicals; speed of rotor 5 m/s, 4 cycles, 40 seconds per cycle). The homogenate was decontaminated using equal volume (1:1 ratio) of 4% NaOH for 5 minutes and neutralized using phosphate-buffered saline (pH 7.4). The decontaminated samples were inoculated to BACTEC 960 Mycobacteria Growth Indicator Tubes (MGIT) with BBL MGIT PANTA antibiotic mixture supplement (Becton Dickinson Diagnostic Systems, Sparks, Maryland). Additionally, 100 µL of the sample was inoculated onto 2 Lowenstein-Jensen (LJ) slopes, 2 LJ slopes with sodium pyruvate supplement (LJ-SP), and 2 McCartney bottles containing 5 mL selective Kirchner media (SK). Smears of the processed deposit were prepared and stained using the Ziehl-Neelsen method (1% carbol fuchsin, 25% H_2_SO_4_, 0.1% methylene blue).

The MGIT cultures were incubated for 7 weeks, and LJ and LJ-SP slopes for 8 weeks. After incubation for 6 weeks, SK cultures were decontaminated using the modified Petroff method using an equal volume of 4% NaOH (1:1 ratio), incubated for 5 minutes, and further inoculated onto LJ and LJ-SP slopes and incubated for an additional 8 weeks. This additional decontamination step ensures proper recovery of MTBC isolates, given the high contamination rates typically encountered with slaughterhouse-derived tissue samples. When colonies appeared on solid media or if growth was observed in MGIT tubes, immunochromatographic assays were performed to confirm the presence of MTBC.

### Histopathology

A portion of the remaining tissue samples were fixed using 10% neutral buffered formalin and processed by routine paraffin-embedment techniques. The tissue blocks were sliced into 5-mm sections and stained with hematoxylin and eosin. The histological gradings (1–4) were based on the appearance of granulomatous epithelioid cells with or without multinucleated giant cells accompanied with or without necrosis (including caseous necrosis and liquefied necrosis) [20].

### Genotypic Confirmation Using Polymerase Chain Reaction

DNA was extracted from cultures using the cetyltrimethylammonium ammonium bromide/sodium chloride method. The DNA isolated from the samples was subject to polymerase chain reaction using MPT64 (10 pmol/µL) and IS6110 (10 pmol/µL) primers to ascertain whether the cultures belong to MTBC. The forward and reverse primers (FP and RP, respectively) for the 240 bp MPT64 gene were FP- 5′-TCCGCTGCCAGTCGTCTTCC-3′ and RP- 5′-GTCCTTCGCGAGTCTAGGCCA-3′, whereas those of the 123 bp gene IS6110 were FP- 5′-CCTGCGAGCGTAGGCGTCGG-3′ and RP- 5′-CTCGTCCAGCGCCGCTTCGG-3′.

### Whole Genome Sequencing and Analysis

Paired-end WGS was performed on the isolated DNAs. Fragmented DNA libraries were constructed using the Nextra XT DNA library preparation kit (Illumina) and sequenced on a HiSeq 2500 (Illumina) instrument. Raw sequences were processed through Kraken2 (version 2.1.1) (github.com/DerrickWood/kraken2) to assign taxonomic labels and check for contamination. Genome sequences were analyzed using the vSNP pipeline to identify and validate single-nucleotide polymorphisms (SNPs) and produce annotated SNP tables and phylogenetic trees [21]. In brief, filtered reads were aligned to the reference genome *M tuberculosis* H37Rv (NC_000962) using the BWA-MEM algorithm (version 0.7.17.1). SNPs were called using Freebayes (version 1.3.1) [22] and verified using IGV [23]. SNP distance between the isolates was calculated using SNP-dist (version 0.8.2) (https://github.com/tseemann/snp-dists). Phylogenies were constructed using RAxML (version 8.2.4) with a GTRCATI model of substitution and a maximum-likelihood algorithm with 1000 bootstrap replication. Tree visualization, annotation, and editing were performed using the integrated Tree of Life (iTOL) (version 6.5.1) [24]. RD-Analyzer was used to infer species and lineages based on the presence or absence of region of difference (RD) [25]. RDScan was utilized to identify large deletions and putative RDs within isolates [26], and TB-profiler used to predict genotypic resistance in the isolates [27].

### Drug Susceptibility Testing

DST was performed using the BD BACTEC MGIT 960 SIRE Kit (Becton Dickinson Diagnostic Systems) with standard first-line drugs. Lyophilized drugs were reconstituted in sterile distilled water, and 0.1 mL of each reconstituted drug was added into separate MGIT tubes to achieve the following final concentrations: streptomycin, 1.0 µg/mL; isoniazid, 0.1 µg/mL; rifampin, 1.0 µg/mL; and ethambutol, 5.0 µg/mL. For samples resistant to first-line drugs, DST was also performed for pyrazinamide, 100 µg/mL; levofloxacin, 1.0 µg/mL; linezolid, 1.0 µg/mL; clofazimine, 1.0 µg/mL; bedaquiline, 1.0 µg/mL; delamanid, 0.06 µg/mL; and moxifloxacin at both 0.25 µg/mL and 1 µg/mL. The test culture was prepared in 1:5 dilutions with saline, and 500 μL of culture was added to the MGIT tube. Drug sensitivity was determined by comparison of culture growth with growth control tube as per the manufacturer's instructions.

## RESULTS

### Samples and Mycobacterial Culture and Histopathological Findings

The 567 samples comprised a total of 280 bronchial lymph nodes, 202 mediastinal lymph nodes, 37 cranial lymph nodes, 21 liver samples, 4 lung samples, 3 mesenteric lymph nodes, 18 prescapular lymph nodes, 1 ileocecal lymph node, and 1 spleen sample (Supplementary Table 1). Nontuberculous mycobacteria were identified in 116 samples. Among 567 samples collected from 500 animals, 61 exhibited gross VLs. A χ^2^ test of independence was performed to examine the relation between VLs and recovery of positive MTBC samples. The relation between these variables were significant (χ^2^ [2, n = 567] = 77.4891; *P* < .00001). Lymph nodes with VLs were significantly more likely to be positive for MTBC compared to lymph nodes without VLs (Supplementary Table 2).

Tissues recovered from 9 of the 500 animals (18 per 1000 animals [95% confidence interval {CI}, 6–29]) were positive for the presence of acid-fast bacilli using Ziehl-Neelsen staining, while 16 of 500 animals (32 per 1000 animals [95% CI, 16–47]) tested positive for MTBC by culture. Histopathological analysis revealed stage 1–4 granulomas in the study samples (Supplementary Figure 1, Table 1).

**Table 1.** Culture and Drug Susceptibility Profiles of Lymph Node Samples From Cattle at Slaughter Positive for *Mycobacterium tuberculosis* Complex

Sample No.	Lab Number	Tissue	Smear^a^	Solid Culture^b^		MGIT^c^	*Mycobacterium* Species^d^	Drug Susceptibility Testing^e^												Histopathology Grading^f^
				LJ	SP			S	H	R	E	LX	MX (0.25)	MX (1)	LIN	CLOF	BDQ	DLM	PZA	
1	KL004	Mediastinal LN	N	N	N	P	*M orygis*	S	S	S	S	NA	NA	NA	NA	NA	NA	NA	NA	1
2	KL012	Bronchial LN	P	N	N	P	*M orygis*	S	S	S	S	NA	NA	NA	NA	NA	NA	NA	NA	1
3	KL013	Mediastinal LN	P	N	N	P	*M orygis*	S	S	S	S	NA	NA	NA	NA	NA	NA	NA	NA	1
4	KL017	Mediastinal LN	P	P	P	P	*M orygis*	S	S	S	S	NA	NA	NA	NA	NA	NA	NA	NA	1
5	KL040	Bronchial LN	P	N	N	P	*M tuberculosis + M orygis*	S	S	S	S	NA	NA	NA	NA	NA	NA	NA	NA	2
6	KL043	Bronchial LN	P	N	N	P	*M orygis*	S	S	S	S	NA	NA	NA	NA	NA	NA	NA	NA	2
7	KL115	Bronchial LN	P	N	N	P	*M orygis*	S	S	S	S	NA	NA	NA	NA	NA	NA	NA	NA	3
8	KL385	Liver	N	N	N	P	*M tuberculosis*	R	R	R	S	R	R	S	S	S	S	S	R	2
9	KL386	Liver	N	N	N	P	*M tuberculosis*	R	R	R	R	R	R	S	S	S	S	S	R	2
10	KL490	Lung	N	P	P	P	*M orygis*	S	S	S	S	NA	NA	NA	NA	NA	NA	NA	NA	3
11	KL493	Bronchial LN	N	N	N	P	*M orygis*	S	S	S	S	NA	NA	NA	NA	NA	NA	NA	NA	3
12	KL496	Bronchial LN	N	P	P	P	*M orygis*	S	S	S	S	NA	NA	NA	NA	NA	NA	NA	NA	4
13	KL498	Bronchial LN	P	P	P	N	*M orygis*	S	S	S	S	NA	NA	NA	NA	NA	NA	NA	NA	2
14	KL499	Bronchial LN	N	P	P	N	*M orygis*	S	S	S	S	NA	NA	NA	NA	NA	NA	NA	NA	1
15	KL536	Mediastinal LN	N	P	P	P	*M orygis*	S	S	S	S	NA	NA	NA	NA	NA	NA	NA	NA	4
16	KL541	Mediastinal LN	N	P	P	N	*M orygis*	S	S	S	S	NA	NA	NA	NA	NA	NA	NA	NA	4

The table presents culture, phenotypic susceptibility testing results, and histopathology gradings for the isolates recovered from tissue samples of cattle processed at a slaughterhouse in Chennai, India. Each isolate is identified by a unique identifier, and the type of tissue sampled is noted.

Abbreviations: LJ, Lowenstein-Jensen media; LN, lymph node; MGIT, Mycobacteria Growth Indicator Tube; N, negative or no growth; NA, not applicable/not performed; P, positive; R, resistant; S, susceptible; SP, sodium pyruvate–supplemented.

^a^Results of the Ziehl-Neelsen stain for the presence of acid-fast bacilli, with “N” indicating a negative result and “P” indicating a positive result.

^b^Growth results on LJ media and SP media, with “N” for no growth and “P” for positive growth.

^c^“P” positive growth in Mycobacteria Growth Indicator Tubes and “N” indicating no growth.

^d^The species of *Mycobacterium tuberculosis* complex identified in each isolate.

^e^Drug susceptibility testing results are provided for a range of first-line and second-line antitubercular drugs, with “S” indicating susceptibility, “R” indicating resistance, and “NA” indicating that the test was not applicable or not performed for that drug. The drugs tested include streptomycin (S), isoniazid (H), rifampin (R), ethambutol (E), levofloxacin (LX), moxifloxacin at 0.25 µg/mL and 1 µg/mL (MX), linezolid (LIN), clofazimine (CLOF), bedaquiline (BDQ), delamanid (DLM), and pyrazinamide (PZA).

^f^The histopathology findings have been graded from 1 to 4 based on the appearance of the stained tissue. Stage 1 granulomas consist of collections of epithelioid macrophages accompanied by small quantities of lymphocytes, granulocytes, and multinucleated giant cells. Genomic analysis: Stage 2 granulomas contained infiltrates of epithelioid macrophages, lymphocytes, and multinucleated giant cells and had variable degrees of central necrosis with a thin connective tissue capsule. Stage 3 granulomas were composed of a central necrotic core, surrounded by a zone of macrophages admixed with lymphocytes and multinucleated giant cells. Portions of the necrotic core may have been mineralized. Encapsulation of the necrotic core and cellular infiltrate by a complete fibrous capsule distinguished this stage from earlier stages. Stage 4 granulomas were observed as a coalescence of stage 3 granulomas with multifocal necrotic and multifocal partially mineralized centers, surrounded and subdivided by thick fibrous bands.

### Genomic Analysis

An average of 7 million read pairs (range, 3–21 million) were generated for each sample with a mean read length of 182 bp with an average coverage depth of 321 (range, 38–621; median, 136). As assessed by Kraken, the taxonomic profiles of all the isolates revealed relatively low (<7%) levels of non-MTBC contamination. The sequence reads were further processed with vSNP and mapped to *M tuberculosis* H37Rv, with resulting genomic coverage ranging from 97% to 99% (Supplementary Table 3). Thirteen of the 16 isolates were identified as *M orygis*, 2 isolates were identified as *M tuberculosis* lineage 2 (L2) Beijing (Pacific), and 1 (KL040) was identified as a mixed sample comprised of approximately 80% *M tuberculosis* sensu stricto and approximately 20% *M orygis* based on lineage-defining SNPs as illustrated in Supplementary Figure 2 [17, 28, 29]. A total of 230 SNPs were identified among the 13 *M orygis* isolates, with pairwise distances ranging from 0 to 123 SNPs (Supplementary Table 4). We identified 2 set of transmission clusters among the *M orygis* isolates, where cluster 1 comprises KL012, KL013, KL017, and KL115 with no SNP difference and cluster 2 consisting of KL004, KL490, KL493, KL496, KL498, KL536, and KL541 separated by 3–20 SNPs. Similarly, the *M tuberculosis* L2 strains KL385 and KL386 also form a cluster with a difference of 7 SNPs.

RD analysis identified the animal-adapted MTBC clade-specific deletions in the regions RD 7–RD10 in all of the 13 *M orygis* isolates, characteristic of MTBC clade La3 [30]. *Mycobacterium tuberculosis* L2-specific deletions (RD105, RD207, RD181), and an additional deletion of RD150 specific for the Pacific sublineage were also observed in 2 isolates (KL385 and KL386) [26]. The distribution of lineage and species-specific RDs among our study isolates is tabulated in Supplementary Table 5.

**Figure 1.**
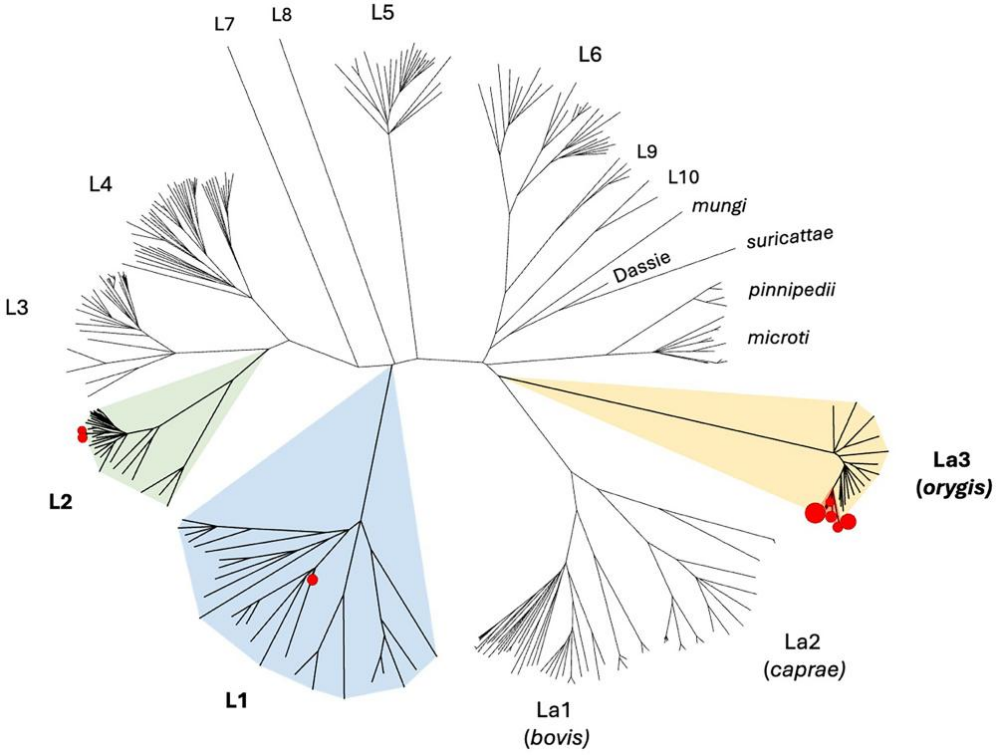
Whole genome sequence–based, maximum likelihood–based phylogeny of representative human-adapted and animal-adapted *Mycobacterium tuberculosis* complex (MTBC) lineages showing placement of the MTBC isolates recovered from cattle during slaughter in Chennai, India (shown in solid circles). The tree is unrooted and branch lengths are shown proportional to nucleotide substitutions between taxa. Human-adapted *M tuberculosis* sensu stricto lineages L1–L10 and livestock-associated lineages La1–La3 are labeled and described as in Zwyer et al [32]. Representative MTBC human and animal-adapted lineages Sequence Read Archive accession numbers are presented in Supplementary Table 6.

**Figure 2.**
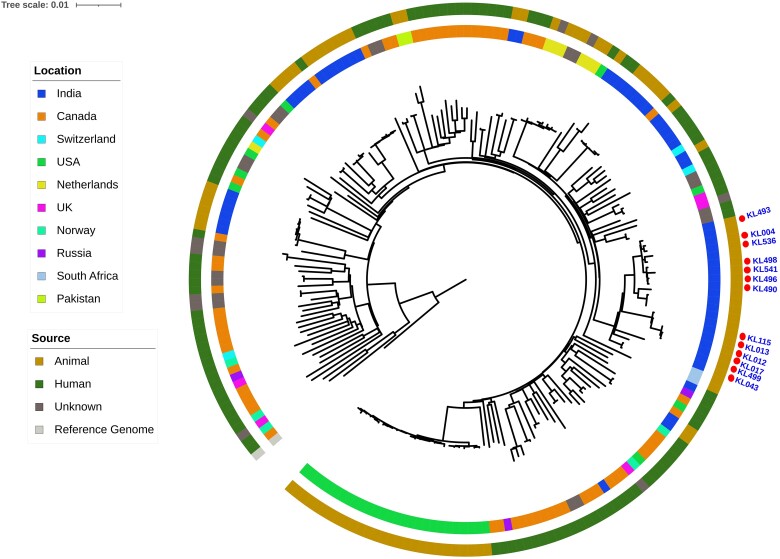
Whole genome sequence–based, maximum likelihood–based phylogeny of representative *Mycobacterium orygis* isolates from human and animals around the globe showing placement of the *M orygis* isolates recovered from cattle during slaughter in Chennai, India. The tree is circular and mid-point rooted and branch lengths are shown proportional to nucleotide substitutions between isolates. Representative *M orygis* Sequence Read Archive accession numbers are presented in Supplementary Table 7. The outer band represents the source and the inner band the location of these isolates.

**Figure 3.**
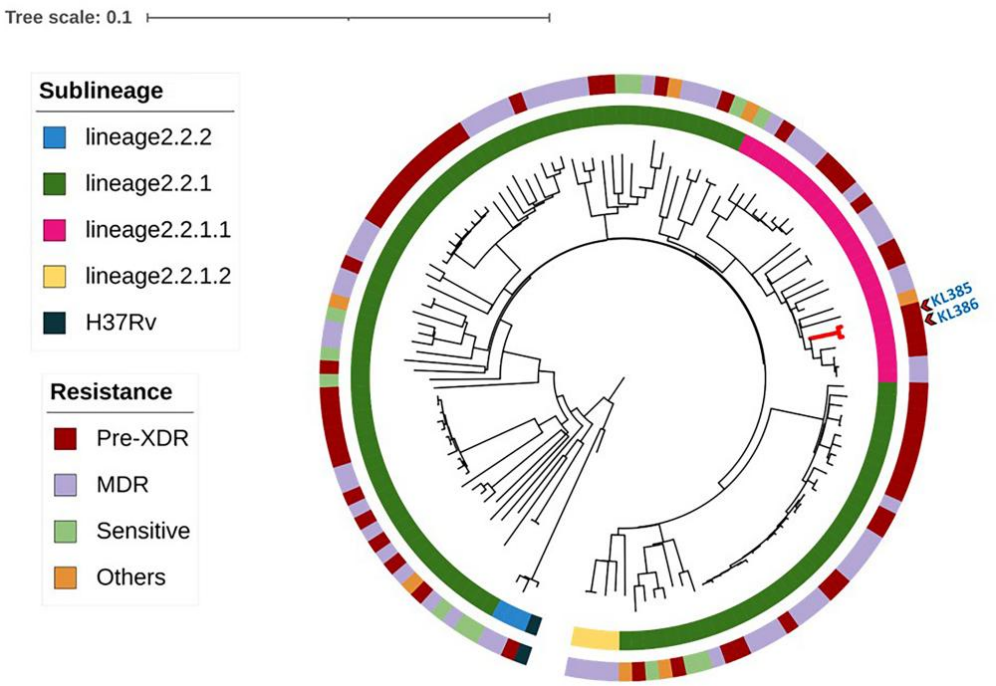
Whole genome sequence–based, maximum likelihood–based phylogeny of *Mycobacterium tuberculosis* lineage 2 (L2) isolates recovered from cattle during slaughter in Chennai, India, placed along with various sublineages of *M tuberculosis* L2 isolates circulating in south India. The tree is circular and mid-point rooted and branch length in proportion with the nucleotide substitutions between the isolates. Representative *M tuberculosis* L2 strains are presented in Supplementary Table 8. The outer circle represents the drug resistance pattern and the inner circle represents the sublineages of these isolates. Abbreviations: MDR, multidrug-resistant; pre-XDR, extensively drug-resistant.

Phylogenetic analyses using maximum-likelihood approaches of the 16 recovered MTBC isolates from the slaughter survey in context with 81 representatives of human and animal origin MTBC lineages from India and elsewhere confirmed that KL040 clustered together with other *M tuberculosis* lineage 1 (L1) isolates and KL385 and KL386 clustered with *M tuberculosis* L2, and the other 13 isolates identified as *M orygis* clustered well with the same lineage (Figure 1; Supplementary Table 6). A phylogenetic tree with 13 *M orygis* isolates recovered from the slaughter survey was also generated in context with 192 *M orygis* isolates available in the public databases recovered from approximately 101 humans and 89 other animals in 10 countries around the globe (Figure 2; Supplementary Table 7). A phylogenetic analysis of the 2 *M tuberculosis* L2 identified in this study was further compared with 198 various sublineages of *M tuberculosis* L2 isolates recovered from humans around the world (Figure 3; Supplementary Table 8)

### Drug Susceptibility Testing

Genotypic DST using TB-profiler identified all 13 *M orygis isolates* and the 1 mixed isolate as sensitive to first-line anti-TB drugs, including streptomycin, isoniazid, rifampicin, and ethambutol. The 2 *M tuberculosis* L2 isolates (KL385 and KL386) were classified as pre-XDR based on their genotypic profiles, with predicted resistance to streptomycin, isoniazid, rifampicin, pyrazinamide, levofloxacin, and moxifloxacin. Notably, although these pre-XDR strains were genotypically predicted to be sensitive to ethambutol, we observed 2 mutations (p.ile450leu & c.1602C > T) in the *embB* gene with uncertain significance to resistance by the World Health Organization [31].

Phenotypic DST results confirmed the genotypic findings except for ethambutol resistance among 2 isolates. The 13 *M orygis* isolates and the 1 mixed isolate were sensitive to the first-line drugs. In contrast, the 2 *M tuberculosis* L2 isolates demonstrated resistance to multiple antimicrobial agents, including pyrazinamide, levofloxacin, and moxifloxacin at a concentration of 0.25 μg/mL and ethambutol at 0.05 μg/mL. These findings confirm that the isolates meet the criteria for pre-XDR based on phenotypic DST results. These results are summarized in Table 1.

## DISCUSSION

Our study reports the prevalence and recovery of *M tuberculosis* and *M orygis* from lymph node samples of slaughtered cattle in Chennai, India. The results show that 16 of 500 sampled animals (32 per 1000 [95% CI, 16–47]) tested positive for MTBC organisms at slaughter, an order of magnitude higher than the 217 per 100 000 humans in 2021 in Tamil Nadu where Chennai is located or the 312 per 100 000 reported in humans across India [7]. This finding highlights a previously underappreciated zoonotic risk that may be posed by these pathogens in this high-burden human and bovine TB–endemic country setting.

Notably, 13 of the 16 MTBC-positive samples were identified as *M orygis*, 1 as a mixed infection of *M tuberculosis* and *M orygis*, and 2 as *M tuberculosis* L2 through WGS analysis. *Mycobacterium orygis* has only recently been recognized as a separate lineage within the MTBC, initially reported as a cause of TB in oryxes and other captive and free-living wild ungulates and from humans, primarily individuals originating from South Asia [29]. *Mycobacterium orygis* was reported for the first time in cattle in India by Refaya et al in 2019 [18]. Another incidence of *M orygis* was reported in a dairy cow in New Zealand, possibly transmitted from an animal handler of Indian origin who had an active TB infection at the time of contact with the cattle [33]. A recent study in Lahore, Pakistan, reported the isolation of 10 *M orygis* isolates and 8 *M tuberculosis* isolates from slaughtered cattle [34]. In most infections with *M orygis*, there is an epidemiological link to South Asia as seconded by the recent report from Alberta, Canada, where all 21 cases of *M orygis* infection were seen in patients of South Asian origin [12]. When *M orygis* was isolated from dairy cattle in Bangladesh instead of *M bovis* (the traditional pathogen), Rahim et al hypothesized that when the human race as we know today migrated “out of Africa,” the dispersion rates of MTBC species across the globe happened at different rates and that the dissemination of *M orygis* could have predated *M bovis* in the South Asian region, becoming endemic to this region [35]. Consistent with these observations, it is hypothesized that *M bovis* might primarily be a pathogen associated with *Bos taurus* breeds in Europe and the Americas, while *M orygis* infects *Bos indicus*–type cattle in South Asia [36].

The interpretation of genomic clustering and its relationship to transmission requires careful consideration of established SNP thresholds. For *M tuberculosis* transmission in humans, Walker et al established that isolates differing by ≤12 SNPs are likely to be epidemiologically linked [37]. In our study of *M orygis* isolates from cattle processed at the same slaughterhouse, we observed 2 distinct clustering patterns: cluster 1 with extremely close genetic relatedness (0–3 SNPs) and cluster 2 with broader genetic diversity (3–20 SNPs). These genomic distances can be contextualized by comparison with a recent *M orygis* outbreak among captive macaques during international transport [38], where isolates differed by 0–13 SNPs despite clear epidemiological linkage. While some pairwise distances in our cluster 2 exceed the conventional 12-SNP threshold, the shared spatial and temporal context suggests potential transmission events, though the precise routes remain undetermined. For the *M tuberculosis* L2 cluster, alternative scenarios must be considered, including the possibility of independent transmission events from humans infected with closely related strains. These findings emphasize the need for integrated genomic and epidemiological approaches when investigating transmission dynamics of MTBC members in cattle populations.

The identification of *M tuberculosis* in cattle is consistent with previous reports where *M tuberculosis* was recovered postmortem from animals and isolated from their handlers, suggesting possible transmission between animals and humans [39]. The strain KL040 was initially predicted as L1 lineage and a detailed examination revealed a more complex picture. Analysis of the BAM file generated by RDscan identified partially filled regions corresponding to L1–specific deletions RD239 (4092080-4092920) and RD147 (1718910-1721212), suggesting the presence of 2 distinct bacterial populations. This observation was further supported by the quantitative analysis of lineage-defining SNPs (Supplementary Figure 2). In brief, we identified characteristic SNPs for both L1 (Rv3915) and *M orygis* (Rv2042, Rv0444c, and Rv1662) with consistent proportions across all loci. The *M tuberculosis* sensu stricto signature dominates at 76%–89% of reads, while *M orygis*–specific variants are consistently present in 11%–24% of reads across different genomic locations. This consistency in proportions across multiple independent loci (with read depths ranging from 64–138 times) provides additional evidence for a true mixed infection rather than technical artifacts, and this represents the first documented case of a mixed infection involving *M tuberculosis* sensu stricto and *M orygis* in cattle. However, since we were unfortunately unable to resample the animal or archived tissue that had all been expended in the initial extraction and culture, we are unable to definitively prove this, and hence are highlighting the potential for coinfection. These findings, if confirmed, may have implications for understanding the transmission dynamics and host adaptation of these mycobacterial lineages.

The identification of pre-XDR *M tuberculosis* L2 in cattle was unexpected and particularly concerning, and to our knowledge, this represents the first report of pre-XDR *M tuberculosis* being isolated from bovine sources. This highlights a major potential public health risk given the documentation of high predisposition of this lineage to transmission and to drug resistance. For instance, in a recent study conducted in India, 24% of *M tuberculosis* L2 isolates from patients with confirmed or suspected MDR-TB disease were found to be pre-XDR [40]. High transmissibility rate of MDR and pre-XDR among *M tuberculosis* L2 isolates has already been reported in Central Asia [41]. Given the known transmission of pre–extensively drug-resistant L2 *M tuberculosis* in South India, along with its isolation from cattle where treatment or exposure to multiple antitubercular drugs is unlikely, it is tempting to speculate that the origin of the infection in cattle is zooanthropogenic. Taken together, the implications of this finding are multifaceted and warrant urgent attention. The presence of pre-XDR *M tuberculosis* in cattle raises concerns about potential bidirectional transmission between humans and animals, which could complicate TB control efforts by introducing drug-resistant strains into the human population through direct contact or consumption of contaminated animal products. Furthermore, cattle may serve as reservoirs for drug-resistant TB strains, potentially allowing for the amplification and spread of resistance genes in the absence of proper detection and control measures. Along with the failure to identify *M bovis*, these observations are consistent with the unique epidemiological landscape of MTBC in this region, underscoring the importance of defining the circulating lineages in both humans and animals in the regional or local context to accurately estimate zoonotic risk.

The presence of *M tuberculosis* in cattle may also lead to diagnostic challenges if bTB is assumed to be caused exclusively by *M bovis* or, as in South Asia, *M orygis*. and highlights the need for species-specific diagnostic tools in both veterinary and human medicine. Additionally, this finding underscores the critical importance of a One Health approach in TB control, emphasizing the need for integrated surveillance and control strategies that span human, animal, and environmental health sectors. In particular, the presence of pre-XDR *M tuberculosis* in cattle highlights food safety risks that may be associated with dairy and meat products, particularly in settings where pasteurization or proper cooking practices may not be universally applied.

For the long term, vaccination strategies also need to be considered. *Mycobacterium bovis*–BCG vaccination strategies are being explored for bTB control in India, supported by new diagnostic tests that detect infected cattle among vaccinated animals [42]. Studies from Ethiopia, where *M bovis* is the predominant cause of bTB, show that BCG vaccination could reduce bTB transmission by up to 74% and prevent 50%–95% of cases over 50 years in high-burden settings [43]. However, the efficacy of BCG vaccination against *M orygis* infection in cattle remains to be determined, particularly in South Asian contexts where this pathogen appears to be more prevalent than *M bovis*. This highlights both the need for safe, efficient, and accessible vaccines to control bTB in endemic regions like India, and the importance of evaluating vaccine efficacy against the locally predominant MTBC members.

It is noteworthy that bTB was a significant animal and public health threat in the early 20th century in many countries, including in North America and Australia, affecting about 90% of cattle herds in Germany and many cattle handlers and children [44]. Rigorous eradication programs, including mandatory tuberculin skin tests, “test and slaughter” policies, and milk pasteurization, drastically reduced bTB incidence. However, these methods are expensive and often unaffordable in low- and middle-income countries with high TB burdens. In these settings, abattoir monitoring serves as a cost-effective passive surveillance technique, crucial for controlling bTB. Detecting lesions during postmortem analysis helps identify bTB prevalence in herds, prompting tuberculin skin tests and appropriate control measures.

This study, despite its relatively small sample size of 500 animals from a single slaughterhouse in Chennai, South India, represents the single largest survey of MTBC lineages present in cattle at slaughter in India. Similarly, while the focused catchment area allowed for an in-depth analysis of the local epidemiological landscape, it remains to be seen how these findings translate to the broader cattle population in Tamil Nadu or other regions of India.

The exclusive sampling of bulls, due to religious and cultural prohibitions on cow slaughter in Tamil Nadu, provided a unique insight into MTBC within the male cattle population, but precluded assessment of infection spread to mammary lymph nodes and potential mycobacterial shedding into milk. This limitation underscores the necessity of future studies to include female cattle to comprehensively evaluate zoonotic transmission pathways. Testing of sampled milk could be another passive way of assessing the prevalence of MTBC in female bovines, though shedding in milk is not always observed [45]. Furthermore, the lack of detailed records on the age and origin of the cattle presented a challenge in thoroughly analyzing epidemiological patterns. However, the application of advanced molecular techniques significantly enhanced our understanding of MTBC lineages present in the sampled population. Finally, although the limited sample size may have constrained the statistical power to detect fewer common lineages, including *M bovis*, our findings provide a crucial baseline for future research.

Future studies with larger, more representative samples and enhanced data collection are needed to confirm these findings and better understand the zoonotic risks and transmission dynamics of TB in cattle populations.

The high burden of MTBC in cattle at slaughter, particularly the presence of drug-resistant *M tuberculosis* and *M orygis*, reveals a critical public health threat requiring urgent action. These findings underscore the immediate need for a systematic surveillance program encompassing both animal and human populations to better understand the risks associated with zTB, especially in regions with close human–animal association and endemic disease. Such surveillance should employ advanced molecular techniques to accurately identify and characterize circulating MTBC strains. The identification of *M orygis* and *M tuberculosis*, but not *M bovis*, as the primary MTBC lineages recovered from cattle emphasizes the need for robust epidemiological investigations to understand transmission dynamics among humans and cattle in India. This complexity demands a One Health approach to effectively address the challenge. Ultimately, achieving the End TB goals will require a comprehensive strategy that accounts for these newly recognized zoonotic risks, highlighting that controlling human TB alone may be insufficient without addressing the animal reservoir.

## Supplementary Material

ofae733_Supplementary_Data

## Supplementary Data

Supplementary materials are available at *Open Forum Infectious Diseases* online. Consisting of data provided by the authors to benefit the reader, the posted materials are not copyedited and are the sole responsibility of the authors, so questions or comments should be addressed to the corresponding author.

## Notes

***Author contributions.*** K. P. conceived and designed this project. H. R., A. K. R., D. K., A. S., A. R., G. R., S. S., and R. R. performed the experiments. K. P., N. R., and M. P. collected the samples from the slaughterhouse. A. K. R., K. P., H. R., T. P. S., and V. K. analyzed the data. N. P. performed histopathological and analysis. K. T. performed the statistical analysis. H. R., A. K. R., V. K., and K. P. drafted the manuscript. All authors read, reviewed, and approved the final manuscript.

***Acknowledgments.*** The help rendered by Mr Ravikumar, Mr Govindarajan, Mr Baskar, Mr Rajaraman, Ms Devi Sangamithrai, Mr Thangaraj Dhanaraju, Ms Angayarkanni Balasubramaniam, Mr Sivaraman, Ms Nithya Ramasamy, and Mr Raghuvaran from the Department of Bacteriology, ICMR-National Institute for Research in Tuberculosis, is duly acknowledged.

***Data availability.*** The sequence reads of the isolates are available in National Center for Biotechnology Information under Bioproject ID PRJNA1091824.

***Financial support*.** This work was supported by the Indian Council for Medical Research (grant numbers ZON/41/2019/ECD-II, Fellowship/97/2022/ECD-II, and ICMR-RA fellowship [2020-6334]). K. P. and V. K. are supported, in part, by the US National Institutes of Health (grant number 1R01A1170753-01A1). V. K. is supported, in part, by the Bill & Melinda Gates Foundation (grant number INV-055079) and the Foreign, Commonwealth and Development Office of the United Kingdom, and received salary support from the Pennsylvania State University (Pennsylvania Agricultural Experiment Station) and the Huck Institutes of the Life Sciences at Penn State.
