# Supplementary material for: Recovery of Mycobacterium tuberculosis Complex Isolates Including Pre–Extensively Drug-Resistant Strains From Cattle at a Slaughterhouse in Chennai, India
Source: Open Forum Infect Dis. 2024 Dec 19;12(1):ofae733. doi: 10.1093/ofid/ofae733 (PMC11736417; doi:10.1093/ofid/ofae733)
Supplement: ofae733_Supplementary_Data [file ofae733_supplementary_data.zip › Appendix.pdf]

1 Supplementary methods:

1.1 Additional genome analysis

In addition to the sequence analysis against *M. tuberculosis* reference genome mentioned in the methodology section, the 13 sequences identified as *M. orygis* were also mapped against the *M. orygis* reference genome 51145 (CP063804) using galaxy-vSNP as mentioned previously. Further *in silico* spoligo analysis was also done for all the isolates in this study using the galaxy-vSNP pipleline.

2 Results

2.1 Additional genome analysis

The genomic coverage of the 13 *M. orgyis* isolates against the reference strain *M. orygis* 51145 ranged from 99% - 100% with quality SNPs ranging between 259 to 2468. The spoligo octal numbers generated for all the 15 isolates except the mixed strain KL040 are shown in the table.

S. No	Sample No.	Total Reads	Q > 30	Genome Coverage (%)	Good SNPs	Spoligo Octal Number
1	KL004	5,481,850	0.94	99.96	281	600300003114671
2	KL012	21,452,146	0.96	99.98	318	600300003114671
3	KL013	17,991,900	0.96	99.99	314	600300003114671
4	KL017	14,707,910	0.97	100.00	312	600300003114671
5	KL043	3,459,464	0.95	99.99	259	600300003114671
6	KL115	7,162,188	0.95	99.98	300	600300003114671
7	KL490	3,163,858	0.81	99.89	294	600300003114671
8	KL493	2,616,668	0.77	99.87	296	600300003114671
9	KL496	2,446,494	0.80	99.86	284	600300003114671
10	KL498	3,207,370	0.79	99.84	300	600300003114671
11	KL499	6,356,478	0.94	99.99	303	600300003114671

S. No	Sample No.	Total Reads	Q > 30	Genome Coverage (%)	Good SNPs	Spoligo Octal Number
12	KL536	6,041,990	0.94	99.96	270	600300003114671
13	KL541	4,969,912	0.90	99.96	2468	600300003114671
14	KL385	NA	NA	NA	NA	000000000003631
15	KL386	NA	NA	NA	NA	000000000003631

2.2 Histopathology

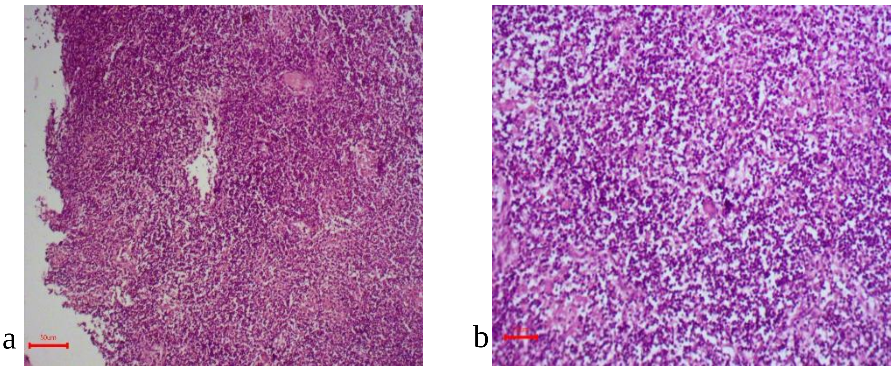

Appendix Figure S1: Histological Characteristics of Tuberculosis Granulomas Across Different Stages in Various Tissues

a & b (KL012, Bronchial Lymph Node): Stage I granuloma exhibiting infiltrates of epithelioid macrophages with a low density of lymphocytes, granulocytes, and multinucleated giant cells. This stage represents the initial immune response, characterized by the formation of an incipient granuloma aimed at containing the infection.

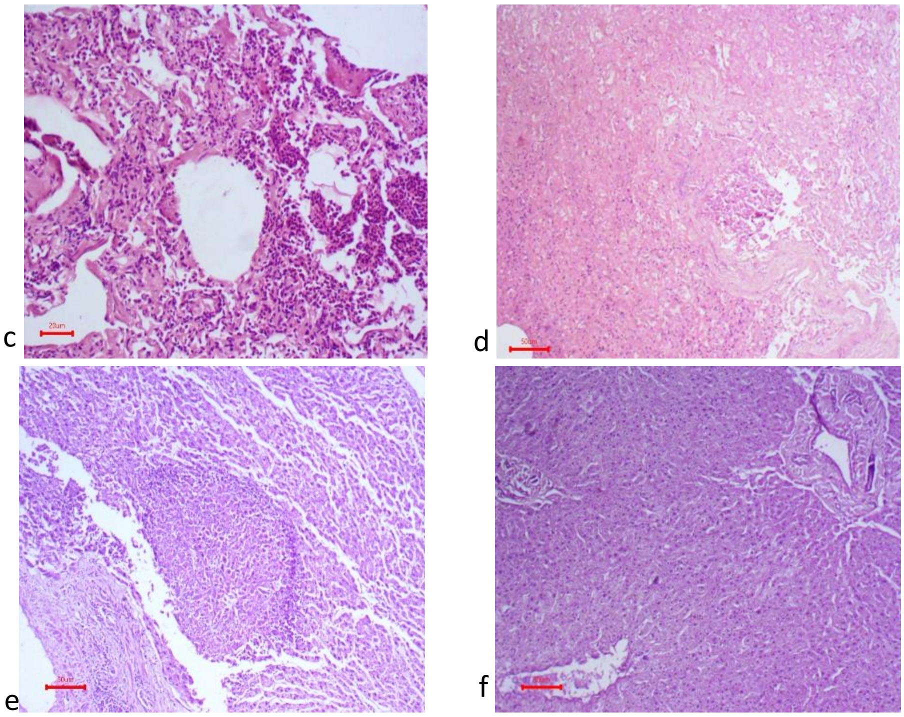

c & d (KL490, Lung): Stage II granuloma displaying a heterogeneous mixture of lymphocytes and interstitial alveolar cells, alongside variable degrees of central necrosis. This stage indicates a progression in the immune response, with increased cellular infiltration and the onset of necrotic tissue as the body attempts to manage the infection.

e & f (KL385, Liver): Stage II granuloma in the liver, displaying epithelioid macrophages and a thin connective tissue capsule. This highlights the systemic nature of tuberculosis, as granulomas can develop in various organs, each exhibiting distinct histological features while reflecting the underlying immune response to the infection.

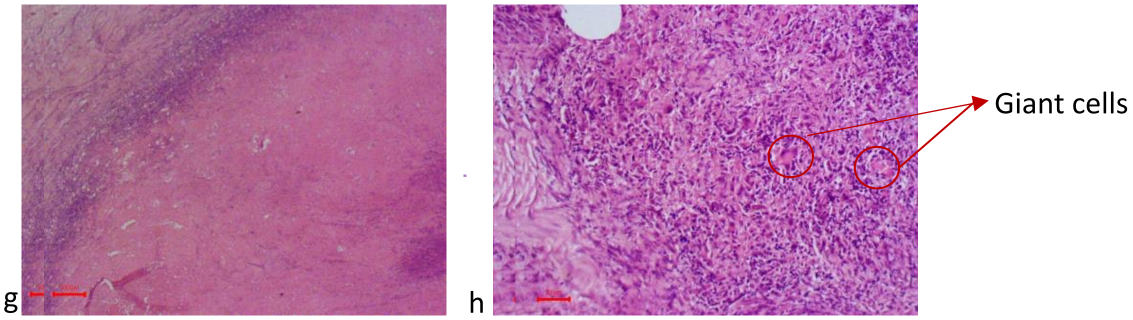

g & h (KL493, Bronchial Lymph Node): Stage III granuloma showing incomplete mineralization of the central necrotic core, featuring multiple giant cells and typical epithelioid macrophages. This stage reflects ongoing immune activity and the beginning of the calcification process, which is critical for containing the infection.

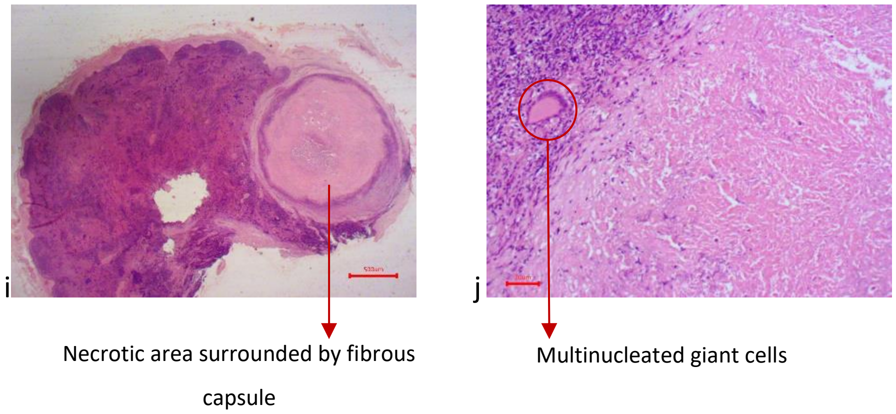

i & j (KL496, Bronchial Lymph Node): Stage IV granuloma characterized by a necrotic area surrounded by a fibrous capsule and the presence of typical giant cells. This mature granuloma represents a well-formed structure that isolates the necrotic tissue, preventing the spread of TB pathogen.

2.3 Mixed strain identification

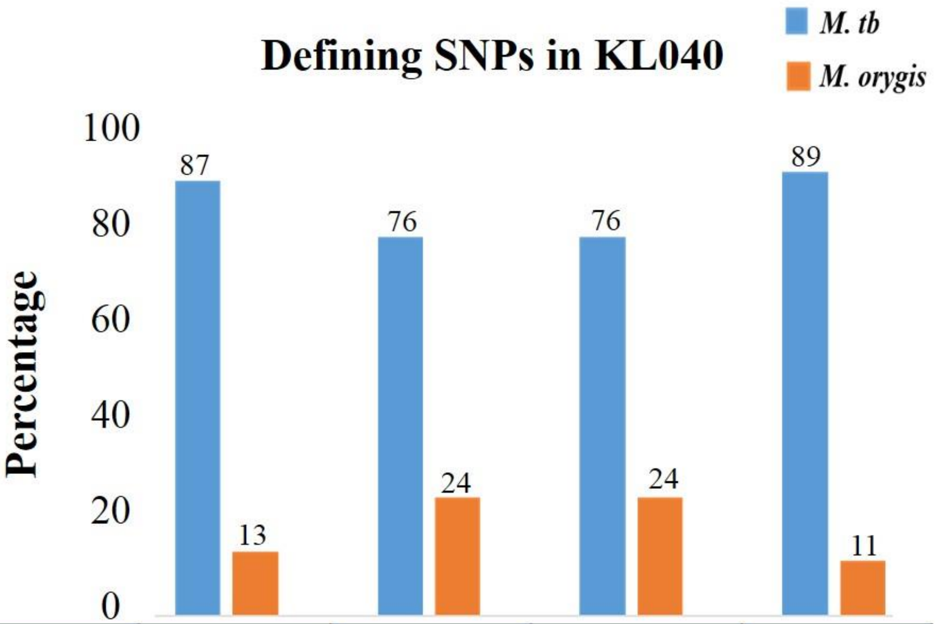

Gene	Rv0444c	Rv2042	Rv3915	Rv1662
Loci	533092	2288569	4404347	1882180
Total counts	79	82	138	64
A	0	62	105	0
C	69	20	0	57
G	10	0	33	0
T	0	0	0	7

Appendix Figure S2: Graphical representation of the defining SNPs of the mixed strain KL040. The Genes representing the lineage specific mutation of *M.orygis* (Rv0444c, Rv2042 & Rv1662) and *M.tuberculosis* L1 (Rv3915) are given in the table with a graph showing the percentage of the two species.
